# High-Throughput Sequencing, a Versatile Weapon to Support Genome-Based Diagnosis in Infectious Diseases: Applications to Clinical Bacteriology

**DOI:** 10.3390/pathogens3020258

**Published:** 2014-04-02

**Authors:** Ségolène Caboche, Christophe Audebert, David Hot

**Affiliations:** 1 FRE 3642 Molecular and Cellular Medecine, CNRS, Institut Pasteur de Lille and University Lille Nord de France, Lille 59019, France; 2 GENES DIFFUSION, 3595, route de Tournai, DOUAI 59501, France; E-Mail: c.audebert@genesdiffusion.com; 3 Transcriptomics and Applied Genomics, Center of Infection and Immunity of Lille, INSERM U1019, CNRS UMR8204, Institut Pasteur de Lille, Univ. Lille Nord de France, Lille 59019, France; E-Mail: david.hot@pasteur-lille.fr; 4 PEGASE-Biosciences, Institut Pasteur de Lille, 1 Rue du Professeur Calmette, Lille 59019, France

**Keywords:** high-throughput sequencing, whole genome sequencing, pathogenomics, bioinformatics, analysis pipeline, P4-medicine, genome-based diagnosis

## Abstract

The recent progresses of high-throughput sequencing (HTS) technologies enable easy and cost-reduced access to whole genome sequencing (WGS) or re-sequencing. HTS associated with adapted, automatic and fast bioinformatics solutions for sequencing applications promises an accurate and timely identification and characterization of pathogenic agents. Many studies have demonstrated that data obtained from HTS analysis have allowed genome-based diagnosis, which has been consistent with phenotypic observations. These proofs of concept are probably the first steps toward the future of clinical microbiology. From concept to routine use, many parameters need to be considered to promote HTS as a powerful tool to help physicians and clinicians in microbiological investigations. This review highlights the milestones to be completed toward this purpose.

## 1. Introduction

Twenty years ago, microbiological research had been radically transformed by the advent of whole-genome sequencing (WGS), which gave rise to the pathogenomics era. More recently, high-throughput sequencing (HTS) has revolutionized sequencing approaches through sequencing platforms and technologies based on multiparallelized shotgun sequences, allowing one to obtain a whole microbial genome in one run or even a fraction of a run. Having access to WGS has significantly increased the knowledge of microorganisms and multiplied the clinical investigations in microbiology. The current standard procedure of clinical microbiology follows a sequential approach, generally consisting of pathogen isolation and in identification before, in some cases, performing drug susceptibility testing and/or epidemiological typing. HTS can be applied in the two major areas of clinical microbiology: diagnostic microbiology (management of infected patients) and the epidemiology of infectious diseases. Obtaining and combining results from time-consuming and heterogeneous methods into a single one is the promise of HTS, which can be foreseen as the future standard tool for a genome-based diagnosis. 

In this review, we will briefly come back on the advent of pathogenomics and the impact of HTS in this domain. Then, the current use of HTS data in genome-based diagnosis will be detailed. Existing current limitations, difficulties and challenges to overcome will be announced and discussed before considering a future HTS integration in clinical microbiology. 

### 1.1. The Advent of High-Throughput Genomics: A Huge Boost for Pathogens Research

In 1995, obtaining the first whole sequenced 1.8 Mb genome of *Haemophilus influenza* [1] required 13 months of work and cost more than 1.5 million U.S. dollars. In February 2014, the Genomes On Line Database (GOLD [2]) included 12, 280 complete bacterial genome projects, and the *de novo* sequencing of a 1.8 Mb genome has a cost of around only ten U.S. dollars. 

**Figure 1:**
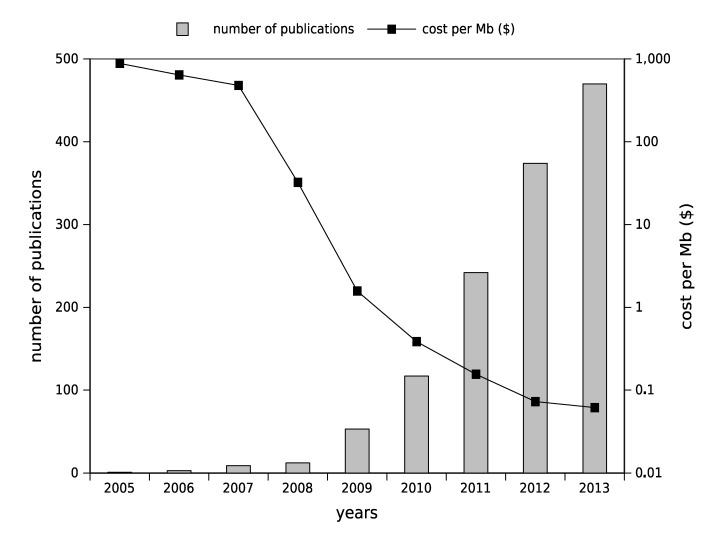
The number of publications associating high-throughput sequencing and clinical microbiology in parallel with raw sequencing cost per year. Cost data from [9] and publication data from PubMed ((“clinical microbiology” OR epidemiology OR outbreak OR pathogen OR virus) AND (“high-throughput sequencing” OR “deep sequencing” OR “next generation sequencing”)).

In 1995, the era of high-throughput genomics began with microarray technologies, which allowed the studying of thousands of genes in parallel. Bioinformatics and statistical approaches have accompanied this technological evolution. In order to take into account the caveats in reliability and reproducibility of the data generated by microarray experiments, the Food and Drug Administration (FDA) decided, in 2005, to launch the MicroArray Quality Control (MAQC) project [3]. In recent years, to circumvent these obstacles, microarray applications have migrated toward sequencing approaches, in such a way that early after the launch of sequencing platforms, a question about the future of microarray technologies has been raised [4]. Be that as it may, the precedent established by the MAQC project likely will facilitate the implementation of suitable tools and processes to standardize HTS applications. With access to microbial whole-genome sequencing data has emerged the concept of genome-based diagnosis [5]. Phenotypic variations of closely related strains can be explained by genome variations (the differences in gene content, point mutations, gain/loss of plasmid, *etc*.). Over the last few years, the rate at which genomes can be sequenced has doubled every 6–9 months [6], and the number of reported clinical investigations that have used HTS has increased exponentially (see Figure 1). The new abundance of available bacterial genomes opens the way to develop new diagnosis and genotyping tools, to unscramble the microbial genomic diversity and to identify and catalog virulence and resistance mechanisms [7]. Recently, many studies have also used HTS to investigate bacterial outbreaks, mainly for the identification of single-nucleotide polymorphisms (SNPs), as this remains the predominantly used polymorphism in bacterial genomic epidemiology [8]. 

### 1.2. High-Throughput Sequencing: Toward Routine Use

The most powerful existing HTS platforms deliver more raw data than required for most clinical investigations (a bacterial genome has a mean size of around only 5 Mb). These platforms could be considered for the moment too slow and too expensive for implementation and use in routine clinical practice. Conversely, the launch of second generation benchtop sequencers, cheaper platforms with performances in terms of throughput more adequate for investigating microbial genomes, could be a better way to enter clinical labs [10]. The difficulty resides in how to handle the knowledge acquired from complete genome collections [11,12]. The early age of HTS soon represented the promise of the exploration of microbial diversity [13], then the dream of accessing the whole microbial genome rapidly became reality and will probably change the practices of diagnosis [14]. Data generated by the wide range of HTS applications and the development of bioinformatics methods and algorithms have increased pathogenomic knowledge since the beginning of the century [15,16]. 

## 2. High-Throughput Sequencing toward a New Medicine?

### 2.1. From PCR-Based Methods for Genotyping to HTS Pilot Studies

In recent years, the evaluation of bacterial genome diversity resulted in the development of PCR-based methods focusing on variable loci to perform a specific detection and/or a genotyping of a pathogen. The terminology “PCR-based methods” aggregates two major approaches that can be distinguished according to their readouts: (i) amplicon or fragment size as the readout, such as multiple-locus variable number tandem repeat analysis (MLVA) [17], multi locus sequence typing (MLST) [18], random amplified polymorphic DNA (RAPD), inter-simple sequence repeat-PCR (ISSR-PCR), clustered regularly interspaced short palindromic repeats-multi-virulence locus sequence typing (CRISPR-MVLST) [19] and (ii) probe-based as readout such as probe-based qPCR, line probe assays (LiPA) and array-based methods. These PCR-based methods can have a commercial formulation and several could be FDA approved or CE-IVD (European Conformity marking for *In-Vitro* Diagnostics) marked and widely used in routine clinical practice (*e.g.*, commercial molecular assays for hepatitis C virus genotyping with the Abbott Real-time HCV Genotype II assay and the Versant HCV Genotype 2.0 LiPA). Furthermore, due to their low cost and great practicability, these genotyping methods are considered for routine use in medical centers [20]. These methods contributed in resolving outbreaks of food-borne pathogen infections, as well as genotyping microorganisms in a clinical infection context. 

When comparing these technologies to sequencing-based methods, many researchers have a critical point of view, arguing that PCR-based methods lack resolution to be able to provide a strain-specific diagnosis and to reach the level of precision enabling one to perceive the supragenome concept *i.e.*, the knowledge of the totality of common and individual-specific genomic material in a bacterial community [21,22]. Recently, various studies have further reinforced this point of view [23,24,25], as PCR-based methods have been shown to be unable to discriminate some closed strains, unlike HTS. In addition, Larrat *et al.* demonstrated that HTS was able to be more sensitive than the Versant hepatitis C virus genotype assay, providing reliable genotype results for 86% of the LiPA failures [26]. Moreover, PCR-based methods for genotyping at the species level require the isolation and growth of infectious strains, which is time consuming and hazardous, especially in a clinical context. This culture of the pathogenic strain is no longer inevitably required with some HTS approaches. Another pitfall of PCR-based methods requires, by definition, specific primer and/or probe sets, which means that the analyzed microbes must have been already encountered. These different difficulties explain why these methods have often been used *a posteriori* by specialized centers or laboratories (*e.g.*, National Reference Centers), too late to be considered as a genome-based diagnosis [14]. PCR-based methods for genotyping can be adapted to HTS [27] by decreasing the cost and increasing the throughput. MLST methods were “a first step in the right direction” [21], but these genotyping methods, unlike WGS, due to the lack of resolution, are not able to provide a strain-specific diagnosis. Providing a whole genome, HTS may rise to the challenge of the supragenome. 

While undoubtedly more information is attainable with sequencing-based methods, in particular, whole genome sequencing (WGS) approaches, workflow and data analysis may currently be considered as complex, cost-consuming and less suitable for routine use. Nevertheless, HTS may overcome the defects of PCR-based methods and may be performed without prior isolation and without an assumption about the identity of the pathogen involved in the clinical picture. 

HTS has been used in different contexts of outbreak and clinical investigation. This kind of platform has a broad spectrum of applications, which can be divided into two classes: culture-dependent or culture-independent approaches (see Table 1). The proof of concept for culture-dependent approaches came in 2012 with feasibility studies in a hospital context on *Staphylococcus aureus* and *Clostridium difficile* outbreaks [28], followed by a pilot study on a multi-drug resistant *E. coli* outbreak [29]. Several examples and comparisons to more standard approaches were then published investigating bacterial genomic epidemiology or, more widely, pathogen evolution [30,31,32,33]. Overall, HTS in a culture-independent strategy leads to a better genotyping resolution and detection of virulence and drug-resistance genes. This information could be exploited by physicians to be interpreted as an *in silico*-antibiogram: from sequences to antibiogram [8]. Investigations on methicillin resistant *Staphylococcus aureus* (MRSA) have demonstrated the relevance of HTS approaches to detect genes involved in the resistome [32,40,41]. Some studies have successfully used HTS assay to detect low-abundance drug resistance mutations (DRM) of HIV [42,43]. HTS has been also used in some fungal infection investigations as an alternative for the identification of fungal pathogens [44]. 

Obtaining a pure culture of pathogens and the phenotypic identification of microbial culture can be time consuming and a source of errors (the difficulty of discrimination between closely related species). Often, *in vitro* culture proves itself slow, difficult or even impossible and can be a real pitfall in cases of outbreaks [21]. Recently, culture-independent strategies have been used more and more, benefiting from the increasing throughput of sequencing platforms to avoid the microbial culture and isolation steps. 

From the community profile, where PCR products are deeply sequenced to identify a microbiota pattern associated with a disease, to single-cell sequencing, where a low quantity of captured pathogens has been amplified with a multiple displacement amplification protocol (MDA) before WGS [38], HTS may be considered an ideal strategy for avoiding pathogen culture and isolation. Recent strategies permitting the capturing of a low number of initial cells have allowed sequencing bacterial DNA directly from a clinical sample [45]. These recent approaches let one predict that culture-independent methods could radically change individual infection management thanks to a genome-based, rapidly available diagnosis, allowing more accurate association between pathogen and symptoms [8,46]. 

Nowadays, benchtop sequencers have been tested in several pilot studies to identify pathogens in the culture-dependent method in the context of clinical investigations [28,29], whereas in the culture-independent method, a sequencing platform delivering higher throughput has been preferred [36,37]. In both approaches, each run of HTS produces a huge amount of short raw sequences, which have to be processed and analyzed with adapted bioinformatics tools. Biological and bioinformatics approaches are inter-related and often complementary in order to efficiently exploit HTS data. For example, host DNA contamination can be discarded after sequencing using the bioinformatic pipeline, but also, with molecular biological methods, before library preparation, to limit this contamination and get more efficient sequencing [47]. 

**Table 1:** Innovative clinical investigations providing findings for a future implementation of high-throughput sequencing (HTS) in genome-based diagnosis. WGS, whole genome sequencing; MLST, multi locus sequence typing.

HTS applications		Study highlights	Pathogens/Sample	Real time/retrospective	Platform	Reference
Culture-dependent	Bacterial genomic epidemiology	feasibility study in a hospital context: improving genetic resolution over common genotyping strategies	*S. aureus*/clinical samples *C. difficile*/fecal samples	real time	Illumina MiSeq	[28]
		pilot study: investigating an outbreak and current limitations for routine use	multidrug-resistant *E. coli*/rectal swab	retrospective	PGM Ion Torrent	[29]
		WGS data exploring MLST: toward a standardized analysis	*C. jejuni* and *C. coli*	retrospective	Illumina HiSeq 2000	[30]
		WGS to rapidly highlight antibiotic resistance determinants	*A. baumannii*/tracheal samples	real time	454-Titanium and Solid version 4	[31]
	Pathogen evolution	high-resolution genotyping by HTS allowing new insights about an emerging pathogen	methicillin-resistant *S. aureus* /clinical isolates	retrospective	Illumina GA IIx	[32]
		Recombination-filtered core genome to understand pathogen adaptation	*E. faecium*/isolates from hospitalized patients	retrospective	Illumina GA IIx	[33]
Culture-independent	Community profiling	proof-of-principle: metagenomics data could be integrated in a diagnosis of cystic fibrosis	airway microbiota in cystic fibrosis/mucolysed sputa	retrospective	PGM Ion Torrent	[34]
		large-scale study monitoring resistance genes in human gut microbiota	gut microbiota	retrospective	Illumina GA IIx	[35]
	Clinical metagenomics and pathogen discovery	a metagenomics approach to avoid pathogen culture and isolation	Shiga-toxigenic *E. coli*/stool samples	retrospective	Illumina HiSeq 2500 and MiSeq	[36]
		an unbiased method to detect viral pathogens	viral pathogens/nasopharyngeal samples	retrospective	Illumina GA IIx	[37]
	Single-cell microbiology	first evidence of a genome capture from a single cell in a clinical context	*P. gingivalis*/sink drain	retrospective	Illumina GA IIx	[38]
		Immunomagnetic separation for targeted bacterial enrichment with multiple displacement amplification	*C. trachomatis*/cervical or vaginal swab	retrospective	Illumina GA IIx and HiSeq	[39]

### 2.2. Bioinformatics to Make Sense of Sequences

HTS technologies are currently able to sequence millions or even billions of bases per run. Once sequences are obtained, the next steps of storing, analyzing and interpreting this huge amount of data requires bioinformatics skills and tools. The cost of sequencing dropped faster than what would have been expected by Moore’s law. Unfortunately, this rapid decrease has not been matched by a comparable decrease in the cost of the computational infrastructure required to mine the data [48]. Every new project in the field will require proportionally less money for the sequencing part, but will have to allocate more resources to the bioinformatics management and analysis of the data. That is the reason why bioinformatics, if not already, will become the bottleneck for a complete and rapid exploitation and interpretation of HTS data. 

The evolution of HTS technologies implied the parallel development of specific and adapted bioinformatics tools. The scientific community makes a lot of tools dedicated to HTS data available (more than 600 tools are listed at [49]). Several tools have to be cleverly linked in order to obtain a functional pipeline to produce final results. Choosing the appropriate tool set, depending on the sequencing technology and the application, can become a real brainteaser. However, some complete and specific pipelines for viral pathogen discovery from HTS data are already available (see Table 2; and for a review, see [50]). 

**Table 2:** Examples of bioinformatics tools used in pathogen studies. I, Illumina; So, ABI -Solid; 4, Roche-454; Hel, Helicos; Ion, Ion Torrent; Sa, ABI Sanger; P, PacBio; N, none; OS, operating system.

**Virus discovery**			
Tool	Features	OS	Reference
CaPSID	Interactive interface to manage, query and visualize results stored in the database	Linux, Mac	[52]
PathSeq	Cloud computingnenvironment	Linux	[53]
READSCAN	Genome relativeabundance	Linux, Mac	[54]
RINS	Identification of non-human sequences	Linux	[55]
VirusFinder	Identification of viruses and integration sites	Linux	[56]
**Mapping**			
Tool	Technology	OS	Reference
BFAST	I, So, 4, Hel	Linux, Mac	[57]
Bowtie2	I, 4, Ion	Linux, Mac, Windows	[58]
BWA -backtrack	I	Linux	[59]
BWA-SW /BWA-MEM	N	Linux	[60]
MAQ	I, So	Linux, Mac	[61]
Novoalign	I, So, 4, Hel, Ion	Linux	
SHRiMP2	I, So, 4	Linux, Mac	[62]
Smalt	I, 4, Sa, Ion, P	Linux, Mac	
**Assembly**			
Tool	Technology	OS	Reference
EULER + Velvet-SC	I	Linux	[63]
IDBA -UD	I	Linux	[64]
MetaVelvet	I, S, 4	Linux	[65]
MIRA	I, 4, Ion, S	Linux, Mac	[66]
Newbler	4	Linux	
SOAPdenovo	I	Linux	[67]
SPAdes	I	Linux, Mac	[68]
Velvet	I, S, 4	Linux, Mac	[69]
**Sequence annotation**			
Tool	Task	OS	Reference
BG-7	Bacterial genome annotation designed for next generation sequencing data	Linux, Mac, Windows	[70]
DIYA	Bacterial annotation pipeline	Linux, Mac	[71]
PROKKA	Annotation of bacterial, archaeal and viral genomes	Linux, Mac	
RAST	Prokaryotic genome annotation service	Linux, Mac, Windows	[72]
RATT	Transfer annotation from a reference genome to an unannotated query genome	Linux, Mac	[73]

In the field of pathogen discovery, bioinformatics tools can be categorized into two groups depending on the application: the pathogen identification and the pathogen characterization. In the case of identification, the aim is to distinguish closed strains in order to rapidly choose a suitable treatment, whereas for characterization, the pathogen genome is studied in-depth in order to highlight some gene transfers and infra-specific variations. 

For both of these applications, a step of mapping against a reference genome is often necessary. Mapping algorithms can be used to localize the reads onto the genome or to filter out reads from the host. A lot of mappers are available: Fonseca *et al.* listed more than 80 mappers [51] (see Table 2 for examples of mappers used in pathogen studies). In the case of pathogen identification, the mapping step is often sufficient to identify the pathogen or a closed strain and to obtain relevant information to choose a treatment. However, the pathogen may need to be better characterized, for example identifying gene transfers or an emerging pathogen. In this case, other bioinformatics tools are required. 

*De novo* assembly algorithms align and merge reads to obtain longer fragments in order to reconstruct the original sequence without a reference sequence. Assembly is useful for studying an emerging pathogen or identifying gene transfers by assembling reads that were not mapped onto a reference genome. Most assemblers are specific to one or a subset of sequencing technologies (see Table 2). In single-cell sequencing, the MDA amplification leads to non-uniform read coverage, as well as elevated levels of sequencing errors and chimeric reads. Some recent assemblers have been developed to deal with these specifications (see Table 2). 

In the field of pathogen discovery, another important task is to annotate the sequences obtained from mapping or assembly, which is often done by comparison, consisting of searching sequence similarity within current databases. For example, PATRIC (PathoSystems Resource Integration Center) is the Bacterial Bioinformatics Resource Center, an information system designed to support the biomedical research community’s work on bacterial infectious diseases [74]. ViPR, the Virus Pathogen Resource, is a publicly available resource that supports the research of viral pathogens [75]. A famous tool for similarity finding is BLAST [76], which is widely used in annotation tools and pipelines. Some annotation pipelines for prokaryotic sequences annotation are also available (see Table 2). In the case of public health applications, identification of specific sequences, such as virulence and resistance genes, is essential to adapt the medical treatment. Drug resistance genes can be detected using BLAST against specific databases, such as ARDB for antibiotic resistance gene screening [77] or using dedicated tool, such as resFinder [78]. For the typing of bacteria species, the MLST scheme [79] can be used to identify sequence type directly from reads or from the obtained assembled sequence. Several free on-line tools exist for the identification of prophage sequences within bacterial genome (*e.g.*, PHAST [80]). For plasmid identification, one possible way is to BLAST the sequence against a plasmid database, such as the PATRIC plasmid database. Another possible analysis is to identify SNPs and DIPs (deletion insertion polymorphisms) suitable for downstream phylogenetic analyses (see [81] for a survey of tools for variant analysis). 

One part of culture-independent sequencing analysis is based on metagenomic approaches and requires specific bioinformatics tools. The analysis of metagenomics data represents a big challenge, as it relies on identifying each individual organism in a mixed sample. Two main metagenomic approaches are used in microbial community analysis: 16S rRNA and whole genome shotgun metagenomics. In 16S rRNA metagenomic approaches, the main step is to assemble overlapping reads and to reduce the dataset complexity by determining operational taxonomic units (OTU) clustering (for example, using UCLUST /USERACH [82] or CD-HIT [83]). In whole genome shotgun approaches, reads are assembled using, for example, MIRA, MetaVelvet or IDBA-UD (see Table 2). In both approaches, the final step is to perform taxonomic classification and compute diversity metrics, which can be done using ARB [84] and the SILVA database [85] or the Greengenes database [86]. Some programs integrate all the analysis steps (*e.g.*, QIIME [87] and mothur [88] for 16S metagenomics and MEGAN [89] for whole genome shotgun metagenomics). 

The analysis of HTS data requires high-performance computational resources. Even if CPU speeds and memory capacities have increased, the huge amount of data to be handled in HTS analyses often requires adequate computational solutions. Several solutions are widely used for HTS programs, such as computer clustering and cloud computing. A computer cluster consists of a set of connected computers with a centralized management approach. With cloud computing, researchers have the option of simply paying for their computing requirements, rather than building and maintaining their own physical computing infrastructure. 

Most academic bioinformatics tools for HTS are technology-dependent, open-source and designed to be run in a UNIX environment with command lines (see Table 2). Computational skills are often necessary, and only a few of them offer a graphical user interface to make them easy to use [90]. To fill this gap, some frameworks have been developed with the famous example of Galaxy [91], an open web-based platform for accessible and reproducible results for genomic research. Some tools are available as web-based resources, which make them easy to use (see Table 2). Another way to make the tools accessible to a broader community is the use of virtual machines (VMs). A VM is a software implementation of a machine (*i.e.*, a computer) that executes programs like a physical machine. A snapshot of a given configuration can be taken and distributed to the community. For example, Cloud BioLinux is a publicly accessible VM containing more than 135 bioinformatics packages, along with documentation, a desktop interface and graphical software applications [92]. 

Bioinformatics is playing and will undoubtedly play a central role in the development of HTS in medicine. One of the many examples showing how bioinformatics impacts the medical management of infectious diseases is the German outbreak caused by the entero-hemorrhagic *O104:H4 E. coli* strain in 2011 [93,94]. The genome sequence was rapidly available through HTS, and at the same time, the microbial characterization, including the clinically antibiotic susceptibility profile, was provided. The authors show that the antibiotic profile can be computationally identified. The German outbreak also used crowd-sourcing as a power tool to fight against pathogens. The genome sequence was released in open-access, and the scientific community was asked for help to annotate the genome. The crowd-sourcing analysis allowed the obtaining of the first annotated version of the genome in a few days. 

The routine use of HTS data in clinical microbiology will depend on the availability of bioinformatics tools, which have to be integrated, *i.e.*, ready and easy-to-use. In addition to bioinformatics solutions brought by the academic scientific community, IVD companies offer and will develop specific bioinformatics tools (most likely proprietary) for these targeted applications. 

With benchtop sequencers, many laboratories and clinical centers can invest in these HTS technologies, whose informatics and bioinformatics is more and more within reach. HTS, as well as the surrounding analytical systems seem to have entered a phase of maturity and are now generalized; nevertheless, some barriers still must be overcome before this (r)evolution finds application in routine genome-based diagnosis. 

## 3. Current Limitations and Challenges

The utility of HTS for genome-based diagnosis in clinical microbiology no longer needs to be proven; the challenge is more, nowadays, in the transfer from pilot studies to routine use in a clinical context. Although HTS is the more powerful method, PCR-based methods currently dominate the market in routine clinical laboratories, because they are more cost-effective, and their workflow and produced data are easier to manage. For the HTS technologies, steps upstream of the sequencing run are not trivial; to prepare a library is an expert user task, and that is why a majority of sequencing platform providers tries to simplify and automate this time-consuming step. Moreover, the newly-arrived HTS methods are facing regulatory hurdles, which may limit their routine use in genome-based diagnosis. 

Currently, HTS is at a technological crossroads: benchtop sequencing has been commercially launched in 2011, between the second and third generation of sequencers. These benchtop sequencers open the way for a wider dissemination; in other words, their compact and easy access format facilitates the generalization of these technologies [95]. Today, technology is still improving capacity, and reliability is constantly evolving, which factually explains the difficulty in adopting and establishing this technology for genome-based diagnosis. The format and the power of sequencing platforms to be used for routine diagnosis have not been totally agreed upon in the community. These specification issues depend on the modality (culture-dependent or culture-independent) of future genome-based diagnosis, on the commercial strategy of sequencer suppliers and on the future certification of these sequencing platforms (FDA and CE-IVD approval). 

According to a survey among physicians, which has tried to enlighten existing barriers for the integration of personalized medicine into clinical practice [96], the access to specific training and clear guidelines were two major items that could influence this adoption of a new clinical practice. A consequence of a young technology, such as HTS, is the lack of academic training, which can result in less adhesion to the new technology. 

High throughput sequencers produce huge amounts of data. Long-term archiving of this data is not a trivial task, and it is evident that the HTS community is facing a storage problem [97]. A reflection on how to store proprietary data concerning patients or research centers has to be made. 

A significant part of HTS analysis is based on the comparison with diverse databases. An improvement of databases used to analyze HTS data in order to obtain well-annotated reference databases is urgently needed [50,98]. Even if some centers organized and distributed some data, a lot of databases exist, and there is a wealth of data, which is left mostly inaccessible and unexplored. Better centralization and data collection should be developed. 

Another limitation with HTS data is the lack of format standardization. Even if some formats are widely used in the HTS field, such as FASTQ , SAM or BAM formats, standardized formats and proce­dures are still lacking. The establishment of standards would help data sharing and connecting tools. In clinical applications, it is also important to integrate and standardize meta-data and include them in the analysis [98]. The integration of results with other types of data, such as the sample collecting place or phenotypic data, is necessary for implementing personalized medicine [99]. Since the technology has been in constant evolution and the algorithms are evolving with it, there is currently no stable pipeline for the analysis of HTS data [100]. Use of a pipeline often implies downloading and installing a lot of software [101], which requires a minimum of computational skills and computational resources, which are limited in a hospital [101]. The lack of an intuitive graphical interface is one of the main limitations in HTS bioinformatics [91]. Indeed, the use of analysis tools is often too complex for most researchers and clinical staff, who choose to use more straightforward approaches, potentially sacrificing the quality of their results [102]. 

In order to be used in hospitals, bioinformatics pipelines have to be trained with benchmarks and rigorously tested on large amounts of clinical samples [50,101]. Indeed, the best practices are still missing in clinical applications. A series of accepted practices for variant discovery is starting to emerge with the 1000 Genomes project [102,103] and has still to be developed for other HTS clinical applications. The lack of standardization in HTS analysis also implies a reproducibility problem. Indeed, very few studies record the exact details of their computational experiments, making it difficult for others to repeat the analysis protocol [102]. Current challenges also include the improvement of general organization, especially between academic and hospital teams, with a global optimization to use findings in their best way. The development of new infrastructures to share data and software creating bridges between these two entities would help the introduction of HTS in clinical medicine [104]. 

In general, regulatory agencies will not approve an IVD that uses a patchwork of ill-defined open source databases and software tools, so this is a requirement to gain regulatory approval for a diagnostic test to be marketed to clinical labs as true IVD. In the future, IVD companies will probably develop proprietary software and databases to address some of these challenges. More likely, in the transition phase, IVD companies will probably form their own “reference labs” and offer these tests and the associated data analysis on a fee for service basis. 

The current challenges and limitations are now known and well described. Some improvements, especially in organization, standardization and data analysis, have to be made in the future to allow the democratization of HTS in clinical applications. 

## 4. A Possible Future

**Figure 2:**
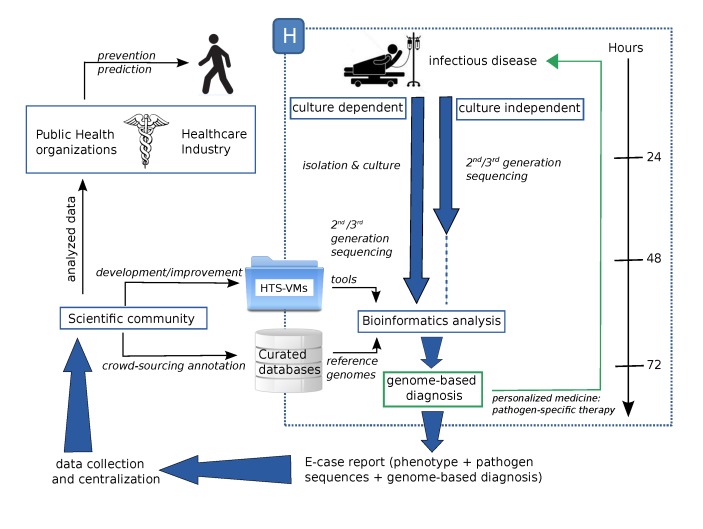
A possible efficient organization of HTS strategies to support a genome-based diagnosis. VM, virtual machine.

To generalize the HTS use for genome-based diagnosis in hospitals, the global data flow has to be improved. Figure 2 shows a possible efficient organization, which could be based on two steps: a first emergency step, in the hospital, and a second step in academic research centers. 

The first step should consist of an emergency stage in a clinical center, leading to a genome-based diagnosis. Sequencing can be achieved by culture-dependent and/or -independent approaches. At this time, it can be difficult to know if HTS will avoid the need for pathogen isolation and culture. Maybe, parallelizing the culture-dependent and -independent methods could be effective for genome-diagnosis, but difficult to manage. Routine use of HTS in order to make a genome-based diagnosis involves standardizing the biological step and the data analysis. These two elements are interdependent and undividable. As much as possible, the whole process must be configured like a “plug and play” box. Experiments performed with high-throughput platforms (*i.e.*, microarrays and HTS) have to be archived following minimum information reporting recommendations, as suggested by work groups, such as MINSEQE [105], in order to adapt data submissions to specific guidelines. Bioinformatics pipelines must be standardized and designed to be easily used by non-experts. One way to achieve this goal is using virtual machines (VMs) [101]. The first benefit of VMs is that they allow the user to be operating system (OS)-independent, which means that, for example, “Windows” users can run tools designed for a Linux environment. VMs can be distributed with pre-installed software, which dismisses the user of complex installation steps. VMs can be used to make collections of HTS tools as a single downloadable unit or to encapsulate analysis pipelines, and they can be designed for cloud-computing or computational clustering. Integrating a graphical user interface with VMs would make them very easy to use. In addition, VMs can be distributed as a “snapshot” of the system with all the data and results, which would also enable researchers to more easily replicate the analysis. The possibility of developing a standardized and clinically-certified HTS-VM could dramatically reduce the complexity of integrating HTS technologies into diagnostic settings, allowing their broader adoption and use. The development of an HTS-VM by a hospital, with the help of a research center, and clinically validated for a specific application, could be downloaded via a specific repository of clinical HTS-VMs and used in another hospital. In addition to HTS-VMs, curated databases would also be available containing a well-annotated reference genome to lead to a genome-based diagnosis. At the end of this first step, which includes sequencing and analysis and which must be efficient and fast, a genome-based diagnosis can be made and a pathogen-specific therapy can be adopted. 

The second step could take place outside the hospital, after the emergency step, producing a genome-based diagnosis. Data produced in the previous step can be exported as an *e*-case report, containing phenotypic data, the genome-based diagnosis and sequencing results (according to the MINSEQE guidelines). This data should be collected and centralized in order to be available for the scientific community. They would be used to improve or develop new HTS-VMs, which would be deposited in a specific repository to be available for all clinical centers. Data would also be used to improve the scientific knowledge about pathogens. For example, with crowd-sourcing, scientists would be able to annotate pathogen sequences and store new knowledge in centralized resources in order to be used in further analyses as reference sequences. Knowledge provided by the intensive study of pathogen sequences about virulence factors and resistance genes could be integrated into healthcare industry R&D projects. This information could be explored to make predictions and provide prevention (*e.g.*, vaccine) for patients. This organization also provides much better options for epidemiology and public health, since each strain can be individually determined, and thus, the actual spread of the pathogen can be traced geographically and in real time. 

Standard clinical laboratories may encounter difficulties in incorporating HTS in their analytical workflow. To overcome these difficulties, some commercial IVD companies have developed strategies using HTS technologies to rapidly identify pathogens, without actually knowing the cause of the infection. For example, Base4 company (Cambridge, U.K.) offers a novel, proprietary nanopore technology for single molecule analysis, pathogen detection and DNA sequencing. Pathogenica (Boston, MA, USA) provides a new technology consisting of probes and a proprietary quantitative pathogen sequencing software package to quantify and identify pathogens. PathoQuest (Paris, France) offers a game-changing approach using the combination of disruptive HTS and bioinformatics/database tools for the discovery and identification of new pathogens. 

The possible organization of HTS strategies to support a genome-based diagnosis, presented in Figure 2, shows an efficient way to entirely use and optimize the data in the context of the P4-medicine (Predictive, Preventive Personalized and Participatory medicine) applied to infectious diseases in order to improve healthcare. 

## 5. Conclusions

*‘Know your enemy and know yourself,* *find naught in fear for 100 battles. Know yourself but not your enemy,* *find level of loss and victory. Know thy enemy but not yourself, wallow in defeat every time’*. This citation from *The Art of War* by Sun Tzu could be adapted to the P4-medicine concept [106] with HTS as a way to understand the interaction between pathogens and their hosts. The P4-medicine concept is currently mainly focused on the host and addresses very few problems applying to infectious diseases. In the concept of P4-medicine, the FDA has granted marketing authorization for the first high-throughput genomic sequencer, Illumina’s MiSeqDx , which will allow the development and use of innumerable new human genome-based tests [107] and will open the way to HTS being used in clinical microbiology. However, combining the knowledge from the host and pathogens together in the P4-medicine concept should change the practice of clinical microbiology in the reference laboratories and should improve the diagnostic step in hospitals. 

The PCR-based methods are currently widely used to identify and partially characterize pathogens in a medical context. HTS is increasingly used in microbiology research to study pathogen genetic diversity, even if this technology is still considered an experimental procedure for medical applications. In addition, the advent of benchtop sequencers promotes the wide spread of sequencing solutions, targeting the personal medicine market and allowing small laboratories to carry out omics strategy to deal with questions about epidemiology and the genetics of pathogens. These last few years, lots of papers introduced pilot and proof-of-concept studies, which have been employed to decipher the genome of pathogens associated with different infectious diseases. Compared to the results obtained with other genotyping methods, these studies conclude that HTS permits a better resolution and a better pathogen discrimination and can be used as the method of choice for a genome-based diagnosis leading to adapted treatments and prophylaxis measures. It is only a matter of time that the benefits of HTS and the use of this technology to establish a genome-based diagnosis will be widely spread. However, interdependent questions must be solved before HTS becomes an efficient and versatile approach to really support a genome-based diagnosis: first, an organizational plan must be adopted, which, in turn, will imply genome-based approaches (culture-dependent or/and -independent), which, in turn, will imply the choice of sequencing platform, which will, in turn, imply the implemented bioinformatics pipeline. Data analysis and the integration of these data are another major bottleneck impeding the use of HTS as a standardized solution to obtain a genome-based diagnosis for the better care of infectious diseases. The absence of a standardized analysis pipeline and the lack of bioinformatics expertise could be solved by the use of a cloud-computing-based pipeline and virtual machines, providing an analysis solution accessible to a broader community. A virtual machine could be developed according to physicians’ purposes and distributed for a better analysis pipeline homogenization to be able to compare data between several hospitals and to reduce the time devoted to analyses. The implementation of HTS approaches in routine laboratories can be considered arduous. Thus, IVD companies already offer workflow and analytical solutions, which are more and more integrated; these commercial solutions may facilitate HTS approaches to become considered as a powerful method to lead to a genome-based diagnosis. The question is not if HTS will become a routine tool for clinical centers, but when and how. 
